# Midgut volvulus is a rare cause of intestinal obstruction in adults: A case report

**DOI:** 10.1016/j.ijscr.2019.03.029

**Published:** 2019-04-04

**Authors:** Hadi Ahmadi Amoli, Ehsan Rahimpour, Negar Firoozeh, Ali Abbaszadeh-Kasbi, Seyed Ali Jazaeri

**Affiliations:** a Sina Hospital, Tehran University of Medical Sciences (TUMS), Tehran, Iran; b School of Medicine, Tehran University of Medical Sciences (TUMS), Tehran, Iran

**Keywords:** Midgut volvulus, Intestinal obstruction, Midgut malrotation, Case report

## Abstract

**Introduction:**

The incidence of midgut vovulus is rare in adults. However, a significant number of cases were seen in infant and children.

**Presentation of case:**

We report a case of a 34-year-old male who presented to the emergency room with persistent periumbilical abdominal pain without any other symptoms. Contrast-enhanced CT showed clearly the typical finding of midgut volvulus like whirlpool sign, corkscrew sign, and the superior mesenteric vein to the left of superior mesenteric artery. The patient was planned for Ladd’s procedure and emergency laparotomy performed successfully with an uneventful postoperative recovery.

**Discussion:**

Managing midgut volvulus is based on presentation. In symptomatic cases surgery is the treatment, but in asymptomatic cases, treatment is controversial.

**Conclusion:**

Although midgut volvulus is rare in adults, it should be considered as an intestinal obstruction.

## Highlights

• Midgut volvulus is usually diagnosed in first weeks of life, or even, in many cases, before birth.
• Incidence of midgut malrotation among pediatrics is 1 in 500 livebirths.
• Incidence of adult midgut malrotation is reported between 0.0001% and 0.19%.
• This is case rare presentation in which a 34 year old man was presented. With diagnosis of midgut volvulus underwent laparotomy.

## 1 Introduction

Midgut malrotation and volvulus could be developed at any age, although it is more common in the first weeks of life, so 90% of affected patients will be diagnosed before the first year of life. Incidence of midgut malrotation among pediatrics is 1 in 500 livebirths whereas incidence of adult midgut malrotation has been reported between 0.0001% and 0.19% [[1], [2], [3]]. In here, we report a young male case with bowel malrotation complicated by midgut volvulus.

This work is reported in line with the SCARE criteria [4].

## 2 Case presentation

A 34-year old male was admitted to the emergency department of the Sina hospital, complaining of a persistent abdominal pain in periumbilical area specially located in the central abdominal area, lasted for 3 days. The pain was not associated with nausea, vomiting, or oral intake. The patient did not defecate for 2 days, but had a normal gas passage, and had not experienced similar pain. Only he reported loss of appetite.

On physical examination, the patient was febrile (T: 38.6) and hemodynamically stable. Further physical examination revealed a soft but diffusely tender abdomen while the maximum point tenderness was in the periumbilical area, and rebound tenderness was absent. Patient’s rectal examination was fecal. There were no signs or symptoms of peritonitis.

A complete blood count (CBC) demonstrated a hemoglobin level of 17.5 g/dL, total leukocyte counts of 9300 per microliter with %78.9 of Neutrophils count. His liver enzymes were normal, arterial Blood gas analysis was suggestive of metabolic acidosis.

To detect air under the diaphragm, an upright chest radiograph was performed.

**Fig. 1:**
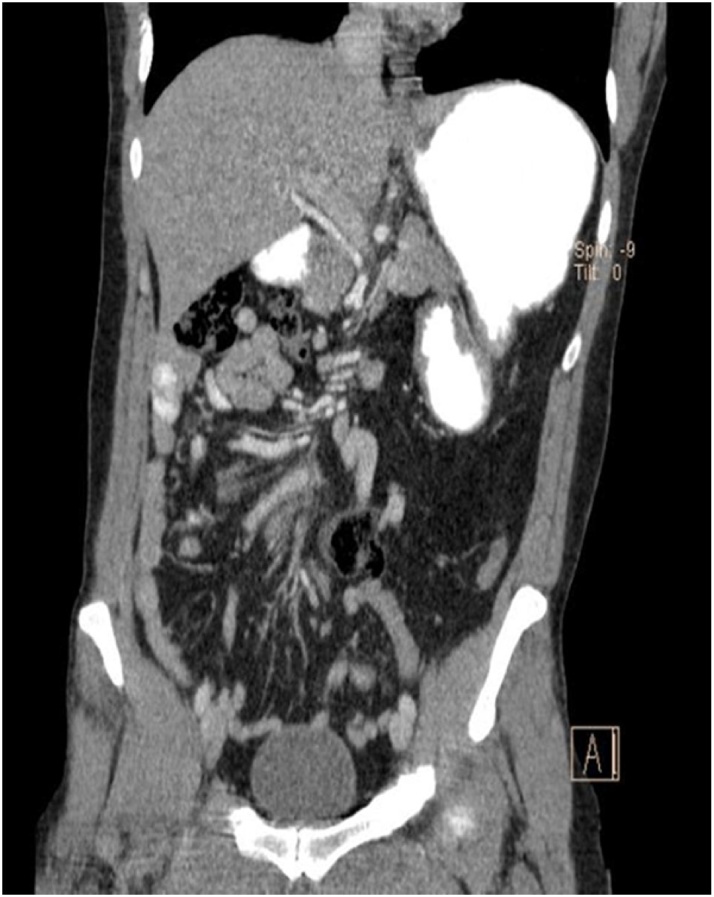
Spiral CT Scan demonstrates midgut malrotation with Volvulus in coronal view.

**Fig. 2:**
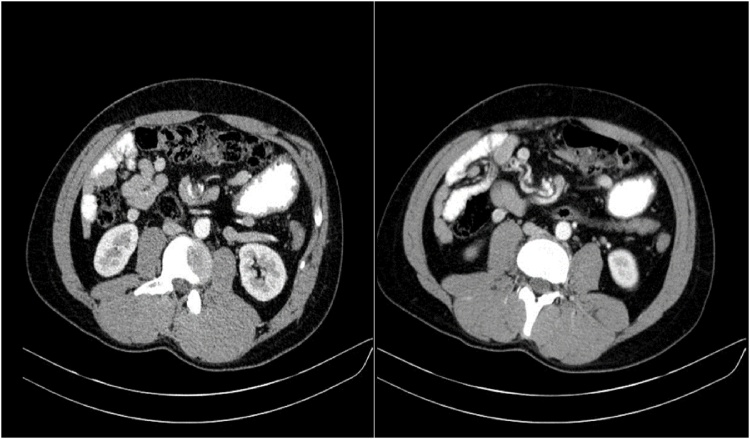
CT showing the characteristic whirpool’s sign.

The abdominal Spiral CT Scan with IV and Oral Contrast demonstrated evidences of midgut malrotation with Volvulus without any obstruction, several enlarged mesenteric lymph nodes—the largest one was up to 9 mm—abnormal position of superior mesenteric vein (SMV)—located at the left side of the Superior mesenteric artery (SMA)— abnormal place of duodenojejunal junction (DJJ), and dispositioned 3rd part of the duodenum (D3), located in front of Superior mesenteric artery and vein (Fig. 1). The characteristic whirpool’s sign was clearly seen around superior mesenteric artery (Fig. 2).

According to the probable diagnosis, patient was prepared for an exploratory laparotomy following adequate resuscitation with intravenous fluids and inserting nasogastric tube and folly catheter. Also, prophylactic antibiotics were given prior surgery.

**Fig. 3:**
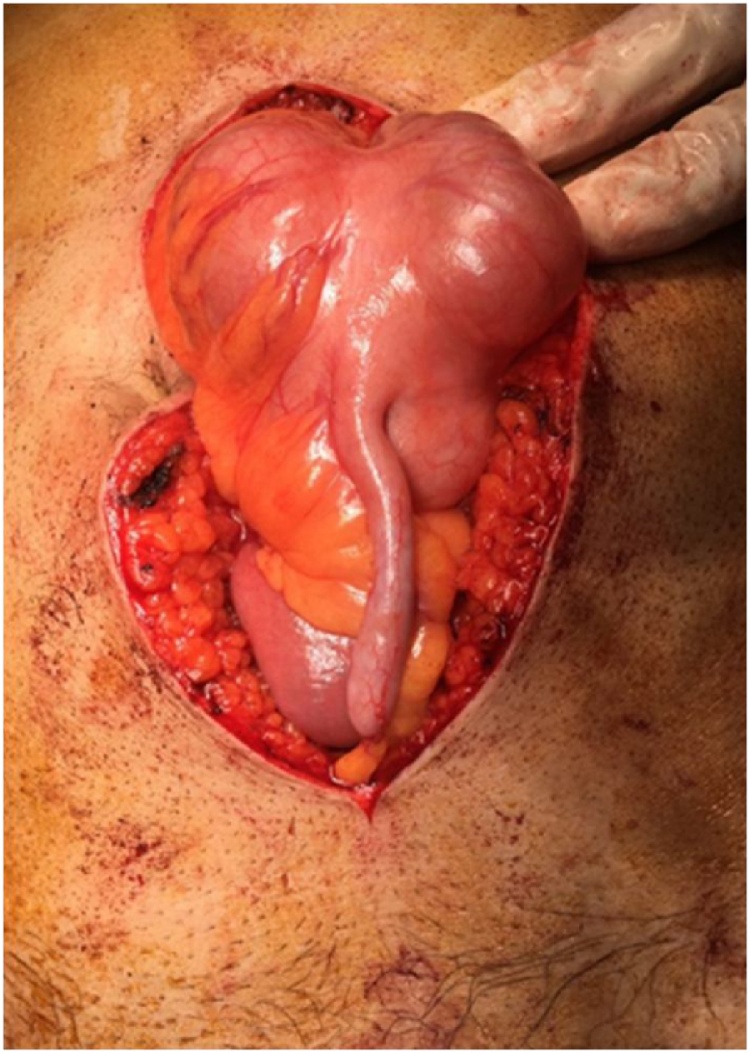
Abnormal position of cecum and appendix.

**Fig. 4:**
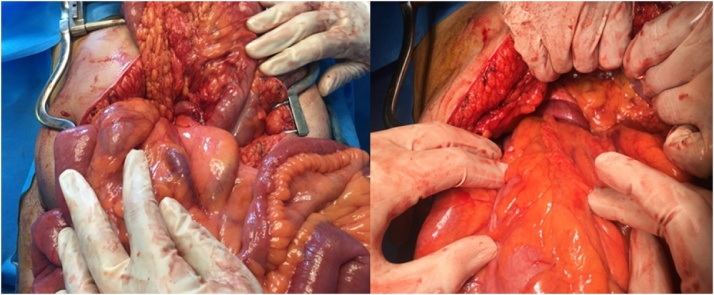
3rd part of duodenum which is passing in front of SMA and SMV.

Midline incision above and below the umbilicus was made upon entrance the cecum and appendix were seen at the midline. There were numerous bands between the bowel loops and abdominal wall. Because volvulus is clock wise, we untwisted it counter clock wisely. The intestine was not gangrened, and only was edematous, improved by heat. Then, adhesion bands between cecum, abdominal wall, duodenum and terminal ileum were released to restore normal alignment. Finally, appendectomy was performed to prevent misdiagnosis in future (Fig. 3, Fig. 4).

The patient was stable postoperatively and had a benign postop course and discharged on the 5th postoperative day.

## 3 Discussion

The first symptom of intestinal malrotation in infants is usually biliary vomiting, and intestinal malrotation should be excluded in all infants with biliary vomiting, particularly those with biliary and irritable vomiting, and if left untreated, vascular disorder may cause bloody stools. Symptoms of progressive ischemia are edema and abdominal erythema, leading to shock and death. Due to the fact that the symptoms are mild in the early stages, this problem should be considered quickly [5].

However, symptoms of midgut malrotation in adults is non-specific as is in infants. Adults with midgut malrotation may be asymptomatic or symptomatic. Those who are symptomatic could have either acute or chronic presentation. Diagnosing the condition is usually difficult because of rarity of the disease [1,6]. Patients with acute onset midgut malrotation more often present with acute bowel obstruction with or without previous symptoms, but most of adult patients, between 80–85%, with midgut malrotation present with chronic symptoms, usually lasting more than 6 months, and they typically complain of intermittent abdominal pain, bloating, and vomiting [1,7].

Treatment of midgut malrotation is based on the presenting type. In acute onset ones, if volvulus is present, emergency laparotomy should be considered immediately to prevent ischemia, but in patients with chronic midgut malrotation that Ladd procedure is used in children and not all aspects of the procedure are appropriate in adults. When patient present with incidental malrotation, decision, either surgical management or conservative management, for managing patient has remained controversial [7,8].

A surgical decision was made for described patient because he had developed volvulus and required emergent laparotomy. The procedure was performed by first performing laparotomy followed by cutting the bands between the cecum and the abdominal wall and between duodenum and the end of the ileum, to release the upper mesenteric artery, and appendectomy was performed. No attempt is made to suture duodenum or cecum in their place. When there is developed ischemia, the volvulus is opened, but the Ladd procedure is not performed and 24–36 h later, the second look is performed, which some degree of recovery is usually seen. Usually when we intend to have second look, we close the abdomen with a transparent silo, to see the color of the intestines and determine the time of reoperation. Completely necrotized areas are resected in the second surgery.

Outcome is highly dependent to patient’s conditions, so in a case with early diagnosis and treatment, prognosis will be preferable. However, late diagnosis leads to high mortality and morbidity, such as short intestinal syndrome [9,10]. Recurrent volvulus is also likely with the incidence of 1.8%–8% [11].

In conclusion, we have presented an adult with acute onset midgut malrotation complicated by volvulus, then he underwent emergent laparotomy to prevent necrosis. Following the surgery, he had a benign hospitalization course, and at follow up visits, he had not any complications.

## Conflicts of interest

None.

## Sources of funding

None.

## Ethical approval

Ethical approval is not applicable for this type of study.

## Consent

Fully informed consent was obtained from the patient.

## Author contribution

Dr Ahmadi Amoli, Dr Rahimpour performed surgery, and all Authors have contributed equally in writing article as well as editing.

## Registration of research studies

This is a case report, and then registration number is not applicable.

## Guarantor

Dr Rahimpour is the guarantor of this work.

## Provenance and peer review

Not commissioned, externally peer-reviewed.
